# Do Parental Education-Related Inequality Matter in Child and Adolescent Utilization of Mental Health Services: Results From a Norwegian Register Linkage Study

**DOI:** 10.1177/11786329211055302

**Published:** 2021-12-10

**Authors:** Tormod Bøe, Mari Hysing, Kristin G Askeland, Jens Christoffer Skogen, Ove Heradstveit

**Affiliations:** 1 Department of Psychosocial Science, Faculty of Psychology, University of Bergen, Bergen, Norway; 2 Regional Centre for Child and Youth Mental Health and Child Welfare, NORCE Norwegian Research Centre, Bergen, Norway; 3 Department of Health Promotion, Norwegian Institute of Public Health, Bergen, Norway; 4 Centre for Alcohol and Drug Research, Stavanger University Hospital, Stavanger, Norway

**Keywords:** youth@hordaland, register linkage, CAMHS, equity, access

## Abstract

Equitable access to health care point to equal access to care for those with equal needs, but pro-rich and pro-educated inequities have been documented in specialized mental health care utilization. This study aimed to investigate equity in Norwegian adolescents’ use of child and adolescent mental health services (CAMHS) with regards to parental education levels, using a survey of 10 257 Norwegian 16- to 19-year-olds subsequently linked to CAMHS data from the Norwegian Patient Registry (n = 970 had been in contact with CAMHS). Analyses using concentration indices (*C*) suggested adolescents with parents with lower education levels had more mental health problems (ie, larger *need; C* = −0.032, *P* < .001) and were more in contact with CAMHS (*C* = −0.025, *P* < .001). Regression analysis suggested that CAMHS contact, and number of unique admissions was largely distributed according to need, but participants whose parents had basic education levels were in contact with CAMHS for slightly longer than predicted from their self-reported mental health problems, age, and sex. Results from this study suggested that contact with CAMHS was largely equitable and mostly influenced by need. There was little evidence of parental education-related inequity in access to, and use of, specialized mental health services.

Mental health problems in youth are common, and although estimates vary, it is suggested that up to 20% of children and young adolescents have a psychiatric disorder.^1,2^ Recent studies have also suggested that the prevalence may be rising, especially for girls.^3,4^ Mental health problems have wide-ranging negative impacts on young people’s health, well-being, future educational prospects, employment and earnings,^5,6^ and they account for a substantial and increasing world-wide burden of ill-health.^7^ Furthermore, psychiatric disorders are not transient phenomena; half of the disorders in young adulthood were preceded by psychiatric disorders in childhood^8^ and psychiatric disorders in childhood are persisting and recurring into adulthood^9^ underscoring a great need and potential for early identification and treatment of psychiatric disorders.

Mental health problems are unequally socially distributed. More adolescents in families with lower household incomes and where parents have lower education levels have mental health problems compared to their more socioeconomically advantaged peers.^10^ This inequality in distribution of mental health problems was also recently documented in a large study of Norwegian children and adolescents where the prevalence of psychiatric disorders in children and adolescents from low-income families was about 4 times greater than for children in high-income families.^11^ A social gradient in psychiatric disorders has also been observed for parental education, with more disorders among youth with lower educated parents.^12^

Many children and adolescents are not being treated for their mental health problems.^13^ Studies from the United States have estimated that only half of the children with mental health problems receive mental health services^14^ and in Europe, the proportion of children not receiving treatment is even greater.^15,16^ Adding to this concern are studies showing inequality in access to mental health services,^17^ suggesting that youth from families with lower socioeconomic status both have more mental health problems and inferior access to mental health services.

Equitable access to services suggests that access should be distributed according to need. Universal health coverage is an important step toward achieving equitable access, and countries that pursue equity in health are generally successful at the primary care level,^18-20^ but pro-rich and pro-educated inequalities have been reported for use of specialized health care services in studies of adults,^21,22^ also in Norway.^23-25^ In a national survey conducted in Norway in 2015, however, young adults with poor health and lower education used mental health services more.^26^ In one of the few Norwegian studies of access to child and adolescent mental health services (CAMHS), children from permanent low-income households were also found to utilize services *more* than children from more affluent households,^27^ but since utilization was not seen in relation to need, we cannot determine whether those findings demonstrate equitable or inequal access to CAMHS. In another study, parental occupation was not associated with neither use of community health services nor CAMHS use in a sample of Norwegian 4- to 12-year-olds.^28^

In general, the role of parental education levels in youth mental health-service access is an understudied area. Parents have an important role as a source of support and a facilitator in help-seeking decisions for youth with mental health problems.^29,30^ Furthermore, higher education levels are strongly related to *mental health literacy*,^31,32^ that is, knowledge that support the recognition and management of youth mental health problems.^33^ Health literacy is an essential component in parents’ decision to seek and successfully utilize services^34^ and is consistently identified as such across the literature on predictors of service use,^35^ models of help-seeking,^36^ facilitators and barriers for access to treatment,^17^ and on equitable service use.^37^ Furthermore, in Norway, and other countries where mental health services are free for youth, parental education levels may be a more important determinant for CAMHS-use relative to financial circumstances in the family.

In the current study, we use a register linked survey of Norwegian adolescents to investigate how parental education levels are associated with contact with CAMHS, number of distinct admissions to CAMHS, and duration of contact with CAMHS. And advantage of the survey-linkage design is the availability of register-based information on CAMHS-use as well as survey data on other important variables found to influence need for mental health services, such as symptoms of mental health problems,^35^ age, and sex,^15^ while also accounting for other important variables that predict use of mental health services, such as family composition^38^ and ethnic minority status.^39^

## Methods

### Study setting

In Norway, specialized mental health services are organized under the regional health authority (RHA), and consists of mental hospitals, community mental health centers and privately practicing psychologists and psychiatrists under contract with the RHAs. For children and adolescents, specialized services have traditionally been provided in designated outpatient clinics^40^ and are free for children and adolescents younger than 18.

### Participants

The present study used data from a linkage between a large population-based study and registry-based data and diagnostic profiles from CAMHS. The population-based youth@hordaland survey was conducted in 2012 and included adolescents aged 16 to 19 years living in the Hordaland County in Western Norway.^41,42^ A total of 10 257 adolescents participated in the survey, comprising 53% of the total adolescent population (n = 19 430).

### Procedure

Participants received information about the study per email and 1 hour was used at school to complete the web-based questionnaire. Adolescents not going to school had the opportunity to participate, as they received the questionnaire by mail to their home address. In addition, mental health services and other institutions (eg, child welfare service institutions and inpatient psychiatric hospitals) were contacted to let adolescents from these settings participate. Informed consent was retrieved from all participants prior to the inclusion. As 846 (8.2%) of adolescents did not consent to linkage with official registries, a total of 9411 adolescents from the youth@hordaland survey were available for the linkage with the registry. Of the 9411 youth/adolescents available for linkage, 970 adolescents (10.3%) had received treatment from or been in contact with CAMHS in the period when they were 12 to 19 years old.

The study was approved by the Regional Committee for Medical and Health Research Ethics (REC) in Western Norway (2011/811/REK Vest) and NSD (371974 and 259631). Following the regulations from the REC and Norwegian health authorities, adolescents aged 16 years and older can make decisions regarding their health (including participation in health studies), and thus gave consent themselves to participate in the current study and for the linkage to registries. Parents/guardians have the right to be informed, and in the current study, all parents/guardians received information about the study in advance.

### Instruments

#### Contact, number of distinct admissions, and duration of contact with CAMHS

The Norwegian Patient Registry (NPR) is the official registry on CAMHS use in Norway and includes information on psychiatric diagnoses based on Axis 1 in the International Classification of Diseases (tenth version; ICD-10), data on the treatment provided, and adverse psychosocial conditions (based on Axis 5; ICD-10) for everyone (that had received services). For those who consented to registry linkage, the data from the youth@hordaland study was linked to NPR. The linkage was performed by the registry owner at the Norwegian Directorate of Health and a de-identified dataset consisting of data from the NPR and the youth@hordaland was used in the present study.

Contact with CAMHS was defined as having a registration within the NPR. The majority of contacts with CAMHS in Norway consists of outpatient clinical consultations, such as conversations between professional health worker(s) and the adolescent and/or the family, and/or indirect contact such as co-operation between a professional health worker and the adolescents’ network, but a minority also receive inpatient psychiatric hospital care.^43^ A continuous variable was constructed for duration of contact with CAMHS, which counted the number of months with an active contact with the services. This variable spanned from 1 to 65 (*M* = 11.45; SD = 10.33, median = 8). Also, a continuous variable for distinct number of admissions to CAMHS was constructed, spanning from 1 to 9 (*M* = 1.34; SD = 0.72, median = 1). For example, admission to inpatient hospital care, as well as repeated admissions to CAMHS after terminated treatment, were registered as separate entries of admissions to CAMHS.

#### Demographic variables and parental education level

Sex and date of birth on all participants were retrieved from the personal identification number from the Norwegian Population Registry and was available for all participants of the youth@hordaland sample. Exact age was estimated by calculating the interval of time between the date of birth and date of participation.

Adolescent reported maternal and paternal educational attainment was measured using the response categories: “primary school,” “high school,” and “college/university” and 1 “unknown” category. For the purposes of the current study, parents with “unknown” education level were omitted from analyses (n = 2174). Maternal and paternal education levels were subsequently combined into 1 variable indicating the highest education level in the family, that is, *Basic* (highest level = “primary school”), *intermediate* (highest level = “high school”), or *higher* (highest level = “college/university”). Adolescents also reported whether their biological parents lived together.

#### Self-reported mental health problems

The Strengths and Difficulties Questionnaire^44^ was used by participating adolescents to self-report mental health problems. The SDQ consists of 5 subscales that measure emotional symptoms, conduct problems, hyperactivity-inattention, peer relationship problems, and prosocial behaviors. Scores of emotional symptoms, conduct problems, peer problems, and hyperactivity-inattention problems were added to compute a SDQ Total difficulties score (ordinal α = .86^45^) with a range from 0 to 40 that we used in the current study. The SDQ has previously been validated in the youth@hordaland sample^46^ and has been found to be a good predictor of diagnostic status.^47^

### Statistical analysis

We calculated descriptive statistics of the sample, and the differences between the sample with and without a registration in CAMHS were investigated using chi-square tests for categorical variables and independent samples *t*-tests for continuous variables.

**Figure 1.**
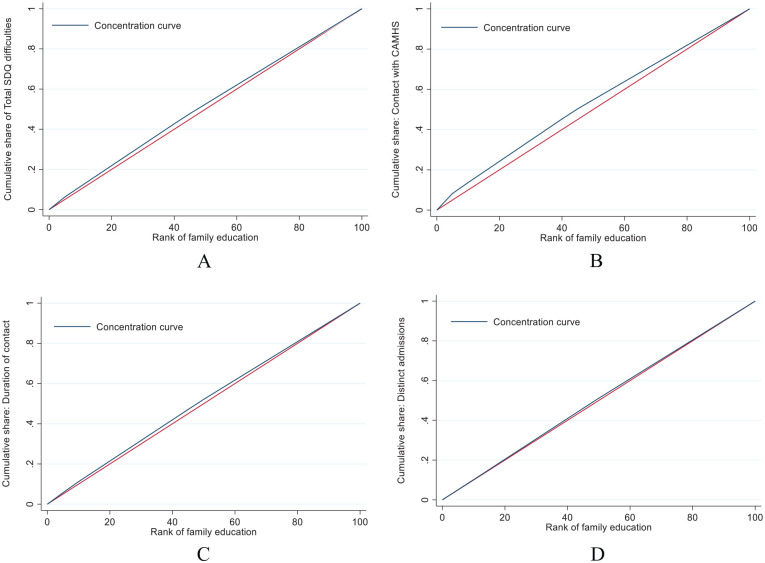
Concentration curves illustrating the cumulative share of mental health problems (A), CAMHS contact (B), duration of contact with CAMHS (C), and number of distinct admissions (D). The blue line is the concentration curve for each variable, the red line illustrates the line of equality. When the blue line is above the red line it suggests inequality in the direction of adolescents with parents with lower education levels having a greater share.

Inequality in contact with CAMHS, duration of contact with CAMHS, and in the number of unique admissions to CAMHS was investigated with concentration curves and concentration indices (*C*) computed with the *conindex*^48^ package for STATA.^49^ The concentration curves illustrate how the cumulative share of the dependent variables (ie, contact with CAMHS, duration of contact with CAMHS, and unique referrals to CAMHS) are distributed across parental education levels sorted from lowest to highest, and the concentration index quantifies deviance from equality. A concentration curve that lies close to the 45° line of equality (see Figure 1) corresponds to a concentration index of 0, which is interpreted as indicating equality. A concentration curve that lies above the line of equality suggests that a larger cumulative share of the dependent variable is concentrated among adolescents with lower parental education levels, which is then reflected in negative values of the concentration index. In contrast, a concentration curve that lies below the line of equality would indicate a higher concentration among those with higher parental education levels.^50^ In the *conindex* package options, we specified all dependent variables as bounded variables with limits and as having true zero-values, and we calculated concentration indices using Erreyger’s correction for bounded variables.^51^

To investigate inequality when accounting for need, we computed registered CAMHS contact stratified by parental education levels and compared it with *need-predicted* and *need-standardized* use. To estimate *need* we regressed CAMHS-contact on age, sex, and self-reported mental health problems in a probit regression model and subsequently predicted the proportion of contact with CAMHS estimated from need (ie, *need-predicted* contact with CAMHS). A similar strategy was used to obtain need-predicted duration of contact with CAMHS and number of unique admissions to CAMHS, but for these analyses we used a Poisson regression model. These 2 latter regression models were fitted to the sample that had been in contact with CAMHS only (n = 970). All regression models were adjusted for ethnicity (own, mother’s, and father’s) and family composition (whether the adolescent lived with 1 or 2 caregivers). To quantify the uncertainty around the difference between registered and predicted contact and use of CAMHS, we calculated 95% confidence intervals for proportions, and used a one-sample *t*-test to measure difference from the registered months and number of admissions and then used the obtained standard error of the differences to compute confidence intervals.

*Need-standardized* contact and duration of contact with CAMHS and number of unique admissions to CAMHS was calculated by subtracting need-predicted CAMHS-contact from registered CAMHS-contact and adding the sample mean of these variables. After standardizing for need, any unequal distribution by parental education levels is suggestive of inequity in CAMHS-contact.

## Results

There were more female than male participants in the total sample, and significantly more female participants had been in contact with CAMHS compared to their male counterparts. Participants in contact with CAMHS were also slightly younger, had a higher relative frequency of parents with basic education level, and they reported more mental health problems. There was also a higher frequency of mothers that were born in Norway, and fewer had biological parents that lived together in the sample with CAMHS contact (c.f. Table 1).

**Table 1.** Descriptive characteristics of the sample.

	Overall n = 9411	No CAMHS contact n = 8441	CAMHS contact n = 970	*P*-value
	% (n)	% (n)	% (n)	
Sex				
Girl	53% (4976)	52% (4390)	60% (586)	<.001^a^
Boys	47% (4435)	48% (4051)	40% (384)	
Age, mean (SD)	17.41 (0.84)	17.42 (0.83)	17.30 (0.83)	<.001^b^
Highest education in family				
Basic	5.2% (389)	4.8% (328)	8.7% (61)	<.001^a^
Intermediate	38% (2873)	38% (2590)	40% (283)	
Higher	56% (4227)	57% (3869)	51% (358)	
SDQ total difficulties, mean (SD)	10.2 (5.2)	9.7 (5.0)	14.1 (5.4)	<.001^b^
Norwegian ethnic origin				
Self	90% (8703)	94% (7814)	93% (889)	>.9^a^
Mother	91% (8499)	91% (7604)	93% (895)	.030^a^
Father	90% (8364)	90% (7502)	90% (862)	.30^a^
Biological parents living together	67% (5957)	69% (5533)	47% (424)	<.001^a^

Abbreviation: SDQ, strengths and difficulties questionnaire.

a Chi-square test of independence for categorical variables.

b *t*-Test for continuous variable.

The concentration curves can be seen in Figure 1.

For mental health problems, the concentration curve lies above the line of equality (see Figure 1, panel A). This suggests that mental health problems were slightly more common among participants with lower educated parents relative to their peers with higher educated parents. This pattern was confirmed by a negative concentration index for mental health problems (*C* = −0.032 [standard error (SE) = 0.03], *P* < .001).

A similar pattern was observed in the concentration curve for contact with CAMHS (see Figure 1, panel B). The concentration curve lies above the line of equity, indicating that participants with lower educated parents were slightly more likely to have been in contact with CAMHS. The concentration index for contact was negative (*C* = −0.025 [SE = 0.007], *P* ⩽ .001) suggesting that CAMHS contact was slightly inequal and that participants with lower educated parents were more in contact with CAMHS relative to their peers with higher educated parents.

Similar concentration curves were obtained for duration of contact (Figure 1, panel C) and number of unique admissions (Figure 1, panel D). For these variables, concentration indices were non-significant, however, suggesting equality in duration of contact (*C* = −0.026 [SE = 0.017], *P* = .137) and in number of distinct admissions (*C* = −0.011 [SE = 0.011], *P* = .302).

The analyses on need-predicted CAMHS contact and use were largely in agreement with the results from the concentration index analysis (c.f. Table 2). For CAMHS-contact, the need predicted proportion need estimate was slightly lower (0.13) than the observed estimate (0.16), suggesting that participants with *Basic*-level educated parents had slightly more contact with CAMHS than what their needs suggested, but the difference was very small and could not reliability be distinguished from 0 (difference [*d*] = 0.003, 95% confidence interval [CI] = −0.019, 0.080). For adolescents whose parents had higher education, results suggested that they received treatment according to their needs. The estimates standardized for need mirrored these results, showing a tendency of more contact among adolescents whose parents had *Basic* education level (Table 2).

**Table 2.** Registered use of CAMHS, duration of CAMHS contact and unique referrals, and predicted and standardized use of CAMHS, duration of CAMHS contact and unique referrals according to needs estimated from self-reported mental health problems, age, and sex.

Parental education level	Contact with CAMHS			
	Registered	Need-predicted^a^	Difference	Need standardized
	Proportion (SE)	Proportion (SE)	P_obs_–P_pred_ [95% CI]	Proportion (SE)
Basic	0.16 (0.018)	0.13 (0.004)	0.03 [−0.019, 0.080]	0.13 (0.018)
Intermediate	0.10 (0.006)	0.10 (0.002)	0.00 [−0.016, 0.016]	0.10 (0.005)
Higher	0.09 (0.004)	0.09 (0.001)	0.00 [−0.012, 0.012]	0.10 (0.004)
Mean (SE)	0.09 (0.003)	0.09 (0.001)		0.10 (0.003)
	Duration of contact with CAMHS^b^			
	Registered	Need-predicted	Difference	Need standardized
	Months (SE)	Months (SE)	M_obs_–M_pred_ [95% CI]	Months (SE)
Basic	12.97 (1.485)	12.16 (0.348)	**0.81 [ 0.128, 1.492]**	12.28 (1.424)
Intermediate	11.70 (0.618)	11.51 (0.162)	0.19 [−0.127, 0.507]	11.63 (0.606)
Higher	10.92 (0.514)	11.24 (0.136)	−**0.32 [**−**0.586**, −**0.053]**	11.12 (0.500)
Mean (SE)	11.41 (0.384)	11.43 (0.100)		11.42 (0.374)
	Number of unique admissions^c^			
	Registered	Need-predicted	Difference	Need standardized
	Number (SE)	Number (SE)	M_obs_–M_pred_ [95% CI]	Number (SE)
Basic	1.38 (0.078)	1.38 (0.018)	0.00 [−0.035, 0.035]	1.35 (0.079)
Intermediate	1.39 (0.047)	1.34 (0.008)	**0.05 [0.034, 0.065]**	1.39 (0.046)
Higher	1.33 (0.039)	1.34 (0.007)	−0.01 [−0.023, 0.004]	1.33 (0.039)
Mean (SE)	1.36 (0.028)	1.34 (0.005)		1.36 (0.028)

All models adjusted for ethnicity (own, mother’s, and father’s) and biological parents living together. Bold text indicate that 95% confidence intervals did not cross 0.

a Coefficients from Probit regression model.

b,c Coefficients from Poisson regression model.

For duration of contact with CAMHS, participants with *Basic* parental education levels had close to 1 month longer observed duration of contact than what was estimated from their needs (*d* = 0.81, 95% CI = 0.128, 1.492). In contrast, adolescents with *Higher* educated parents were in contact with CAMHS for a slightly shorter duration (10.9 months) than what was estimated from their needs (11.2 months, *d* = −0.32, 95% CI = −0.586, 0.053). Standardized for need, adolescents who had parents with higher education level were in contact with CAMHS for a shorter duration than their peers of parents with lower education.

With regards to the number of distinct admissions, it was found that participants with *Intermediate* parental education levels had slightly more observed admissions (1.39) than estimated from their needs (1.34, difference = 0.05, 95% CI = 0.034, 0.065), while differences between participants with *Basic* and *Higher* parental education could not reliably be distinguished from 0. The need standardized number of unique admissions suggested that adolescents who had parents with *Intermediate* education levels required the most admissions.

## Discussion

Using register-linked survey data from Norwegian adolescents, we investigated inequalities in contact with CAMHS related to parental education levels. Contact with CAMHS and duration of contact with CAMHS were largely equitable and corresponding to health needs as defined by self-reported mental health problems, age, and sex. There were some indications of parental education related inequalities in duration of contact, suggesting that adolescents with lower educated parents were in contact for slightly longer than their needs predicted.

The findings with regards to parental education levels are novel in a Norwegian context. They seem to correspond to previous studies showing more use of mental health services by children and young adults with lower SES,^26,27^ although methodological differences in measurements of SES (ie, not measuring parental education) and conceptualizations of need for health services do not allow for direct comparisons. In a small-scale study from the Netherlands, a country with a comparable health care system to Norway, they did not find that educational level of parents impacted mental health service referrals after accounting for adolescent needs.^52^ Relatedly, parental education levels did not influence help-seeking in a sample of 7 to 14 year old children after accounting for sex, presence of father in the home and ethnicity in a sample from the National Longitudinal Survey of Youth and the Child/Young Adult supplement.^53^ The findings from the current study, however, stand in contrast to some previous studies of adults, where higher education has been associated with more use of specialized health services.^23-26,54^

The reason for longer duration of service use when accounting for need among children and adolescents is not certain. It may be partly that this is a result of different disorders across educational levels^55^ or that providing similar levels of care takes longer to do when working with families with lower education levels. For instance, it may be that some conditions or comorbidities that require longer follow-up are overrepresented among children and adolescents with lower education. These differences could also reflect real, and substantial differences related to how health service providers reason about treatment. In families with fewer educational resources, it is possible that treatment is prolonged “just to make sure” it will have lasting effects, while a “you take it from here” reasoning takes place in families with more educational resources. Still, the difference in duration of contact was relatively small, and should not be overinterpreted.

There is uncertainty about to how influential parents were in the adolescent’s help seeking which may be related to the small differences in contact with CAMHS that were documented in this study. In Norway, adolescents older than 16 can seek out and be in contact with help services without parents being informed. Adolescents could therefore have obtained help without involving their parents, potentially lessening the influence of their parent’s education levels. Studies suggest this could be more likely for female participants,^56^ but more commonly, studies report that adolescents experience a range of barriers preventing them from seeking help,^57^ that their parent’s help them overcome.^29,30^

### Strengths and limitations

Among the strengths of the current study is the linking of a large-scale survey and register data, allowing us to report on contact and use of CAMS while accounting for need, as defined by symptoms of mental health problems, age and sex. The SDQ, used for measuring mental health problems has been validated in the current sample^46^ and the score on the SDQ predicts diagnostic status.^47^ A strength of including this instrument is that the total score gives a broad indication of mental health problems, but consequently a weakness might be that it does not cover specific disorders or a measure of functional impact. Age and sex were obtained from personal identification numbers and are accurate. The survey also allowed us to adjust our analyses for other variables known to influence contact with help services, such as family composition and ethnicity.^38,39^

There are certain important limitations that should be considered when interpreting the results from the current study. For one, parental education levels were reported by the adolescents. Previous studies have shown reasonable agreement between adolescent and parental reports of SES-measures,^58,59^ but there could be imprecisions in our parental education variable. There were also many missing responses in the parent education variable resulting in omitting participants from the analyses.

Another potential limitation related to how we conceptualized *need* in the current study. Need for mental health care is particularly difficult to operationalize,^60^ and although our approach is in line with recommended strategies,^50^ our operationalization does not include variables such as functional impairments, nor availability of social networks and sources of social support, which vary with socioeconomic status and may partly explain why some groups need more help from professionals than others.

The present study is limited to mental health service use in specialized health care clinics, in the (free) public health care system. Children and adolescents could also have been in contact with mental health professionals in the primary health care system or purchased services privately as the CAMHS register does not contain this information.^43^ It is more likely that adolescents from families with higher socioeconomic status would pursue private options, as has been shown for private medical services for Norwegian adults.^23-25^

Finally, a limitation could be differences in characteristics between the survey participants who consented or not to registry-linkage which could affect the generalizability of our findings. Previous investigations have shown that those consenting to registry-linkage were slightly older, consumed more alcohol, and had more conduct problems, but there were few differences in the sociodemographic variables that were of particular interest in the current study.^61^

## Conclusion

The results from the current study demonstrated that contact with CAMHS and duration of contact with CAMHS were largely equitable and corresponding to health needs as operationalized by self-reported symptoms of mental health problems, age, and sex in this Norwegian sample.
